# The causal effects of dietary component intake and blood metabolites on risk of delirium: a Mendelian randomization study

**DOI:** 10.3389/fnut.2024.1441821

**Published:** 2024-11-27

**Authors:** Qian Zhu, Yingjian Liu, Xiaona Li, Chao Wang, Zhenyan Xie, Gongjie Guo, Wenqing Gu, Yongzhen Hu, Xiaobing Wei, Yiqi Wen, Yingchao Jing, Shilong Zhong, Li Lin, Xuesong Li

**Affiliations:** ^1^Department of Neurosurgery, The Affiliated Huizhou Hospital, Guangzhou Medical University, Huizhou, Guangdong, China; ^2^Department of Pharmacy, Guangdong Provincial People’s Hospital, Guangdong Academy of Medical Science, Southern Medical University, Guangzhou, Guangdong, China; ^3^School of Medicine, South China University of Technology, Guangzhou, Guangdong, China; ^4^Department of Biobank, Sun Yat-sen Memorial Hospital, Sun Yat-sen University, Guangzhou, China

**Keywords:** blood metabolites, causal effects, delirium, dietary component intake, Mendelian randomization

## Abstract

**Backgrounds:**

Growing evidence has indicated that the nutritional quality of dietary intake and alterations in blood metabolites were related to human brain activity. This study aims to investigate the causal relationship between dietary component intake, blood metabolites, and delirium risks.

**Methods:**

We performed Mendelian randomization (MR) analysis using genetic variants as instrumental variables for dietary component intake, blood metabolites, and delirium. Inverse variance weighting, maximum likelihood, weighted median, weighted mode, and MR-Egger methods were used for statistical analyses.

**Results:**

We found that genetic prediction of salt added to food (odds ratio [OR] 1.715, 95% confidence interval [CI] 1.239–2.374, *p* = 0.001) significantly increased the risks of delirium, while low-fat polyunsaturated margarine used in cooking (OR 0.044, 95%CI 0.004–0.432, *p* = 0.007), cheese intake (OR 0.691, 95%CI 0.500–0.955, *p* = 0.025) and coffee intake (OR 0.595, 95%CI 0.370–0.956, *p* = 0.032) was suggestively associated with decreased risks of delirium. Moreover, increased blood 1-stearoylglycerol levels (OR 0.187, 95%CI 0.080–0.435, *p* = 9.97E-05) significantly contributed to reducing the risks of delirium. 3-methoxytyrosine (OR 0.359, 95%CI 0.154–0.841, *p* = 0.018) also has the potential to decrease the risk of delirium.

**Conclusion:**

Our study highlights the potential causal effect relationships of dietary component intake and blood metabolites on the risk of delirium, which potentially provides novel insights into targeted dietary prevention strategies or biomarkers for delirium.

## Graphical abstract

We performed a two-sample MR analysis to investigate the causal effects of genetically predicted dietary component intake and blood metabolites on the risks of delirium. This study found that a 1-standard deviation (SD) increase in the genetically predicted habit of salt added to food may contribute ~72% increased risks of delirium, while the habit of low-fat polyunsaturated margarine used in cooking, cheese intake, and coffee intake may help to reduce ~31–97% risks of delirium, a 1-SD increase in the blood 1-stearoylglycerol and 3-methoxytyrosine levels may also contribute to reducing ~64–81% delirium risks. Image created with bioRender.com.

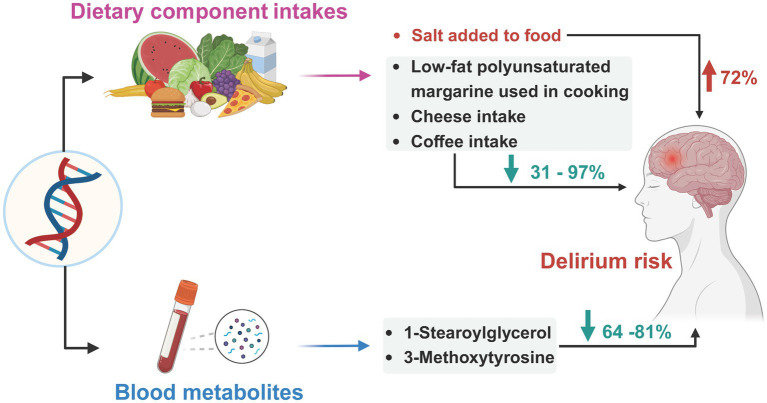

## Introduction

Delirium refers to a brain syndrome, with sudden declined mental status characterized by consciousness disorders, disorganized behavior, and inattention (1, 2). It is a common complication in hospitalized older patients with an estimated incidence of 8–51% (1, 3, 4). It prolongs hospitalization duration and increases mortality and the risk of long-term cognitive impairment (5–7). Delirium is associated with various underlying factors including advanced age, preexisting diseases, metabolic disorders, inflammation, genetic factors, altered sensory function, and so on (8, 9). Although delirium is preventable, most of the current strategies for delirium are primarily used to control the symptoms and treat the underlying conditions, which are less efficacious (10, 11). Therefore, it is urgent to identify potential causal molecular targets or novel preventive strategies for delirium to improve patient outcomes.

Human dietary intake habits and blood metabolites are believed to be closely linked to brain function and mental health. Dietary intake habits have significant or causal influences on the development of most neurological and mental disorders (12–14). Good dietary intake habits such as low-fat, high-fiber diets rich in antioxidants may contribute to preventing and mitigating brain injury and neuroinflammation (13–15). Daily micronutrient intake deficiency also significantly increases the risk of developing delirium (16). Since dietary intervention is modifiable and cost-effective, with higher safety and better compliance, it is likely to be a promising strategy for preventing and managing delirium. In addition, recent observational studies have shown that the serum metabolites including ω3 and ω6 fatty acids, tricarboxylic cycle intermediates, and phosphatidylinositol were linked to the development of postoperative delirium (17, 18). Indole-3-propionic acid was recently reported to protect against the development of postoperative delirium and relieve neuronal damage (19). However, to date, studies on the causal effects of dietary intake and blood metabolites on delirium risks remain to be refined. Performing research on the causal relationship between them could help to provide opportunities for dietary and metabolic interventions.

Mendelian randomization (MR) methods use single nucleotide polymorphisms (SNP) as instrumental variables for inferring causal effects. Since irreversibility of heredity and SNP are randomly assigned at the time of conception, MR can help overcome the limitations of traditional epidemiological or observational studies, including confounding and reverse causation (20). MR is widely used to study disease pathogenesis and potential strategies for prevention and treatment, and they have found that nutrients and metabolic disturbances are strongly associated with neurological disorders (5, 12, 21, 22). The previous MR studies have been extremely reliable in assessing causality, we would like to use it to investigate the association of dietary component intake and blood metabolites with delirium.

## Methods

### Study design

**Figure 1:**
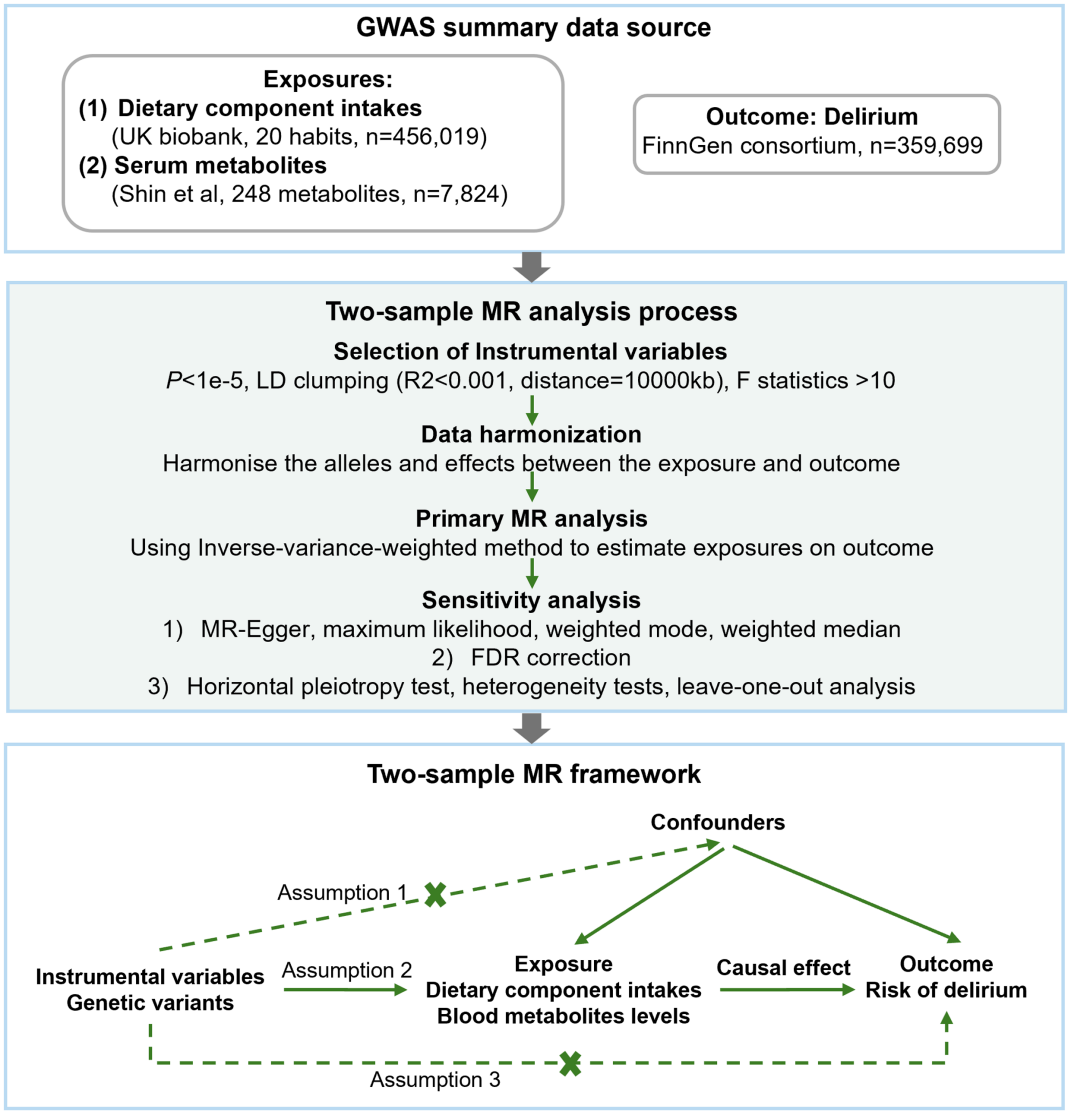
Flow chart of the study design.

The study flow is depicted in Figure 1. Two-sample MR analysis was implemented to assess the potential causal relationship between dietary component intake, blood metabolite levels, and risks of delirium. The MR analysis is according to the following three assumptions to mitigate the bias; (1) instrumental variables (IVs) are strongly associated with the exposure; (2) IVs are not correlated with the confounders; (3) IVs can only affect the outcome through the exposure.

### Data source

Summary-level genome-wide association study (GWAS) data for dietary component intake phenotypes were derived from 456,019 individuals of European ancestry in the UK Biobank (23). Refer to the component intake phenotypes in the study of Zhang et al. (24), we included beef intake, poultry intake, raw vegetable intake, fresh fruit intake, non-oily fish intake, oily fish intake, processed meat intake, pork intake, mutton intake, cheese intake, milk type used (full cream), cereal intake, bread intake, salt added to food, water intake, tea intake, coffee intake, and type of fat/oil used in cooking. During dietary assessment within the UK Biobank study, food and beverage intake over the preceding year was evaluated using a touchscreen food frequency questionnaire (FFQ), while specific foods and beverages consumed with quantities were assessed through a 24-h dietary recall questionnaire (Diet WebQ), which was administered on four occasions. Detailed information about these questionnaires can be found publicly (Data field 113241: Touchscreen questionnaire ordering, validation, and dependencies^1^; Data field 20090: Online 24-h dietary recall questionnaire^2^). The data considered for each dietary pattern encompass both integer variables (e.g., average daily cups of coffee consumption) and categorical variables (e.g., poultry consumption frequency). Unreasonable responses were excluded during data submission.

The summary statistics data for the levels of endogenous metabolites were obtained from a meta-analysis of 7 European populations, as published by Shin et al. (25). The dataset includes a total of 7,824 individuals of European ancestry. Following rigorous quality control procedures, 248 annotated blood metabolites were included.

As for some key metabolites related to low-fat polyunsaturated margarine, summary statistics of plasma concentrations of total fatty acids (Total-FA), omega-3 fatty acids (Omega-3), omega-6 fatty acids (Omega-6), polyunsaturated fatty acids (PUFA), monounsaturated fatty acids (MUFA), total cholesterol (Total-C), total triglycerides (Total-TG), LDL cholesterol (LDL-C), total lipids (Total-L), and HDL cholesterol (HDL-C) are from UK Biobank^3^ (dataset: met-d, *n* = 114,999 ~ 115,078). Blood levels of coffee-related metabolites (caffeine, xanthine, theobromine, and theophylline) are from xenobiotics GWAS by Shin et al. (25) of European populations (*n* = 7,824).

Summary statistics data for delirium were obtained from the ninth edition of the FinnGen biobank, a prospective cohort study involving 359,699 individuals (26). Delirium patients (*n* = 3,039) were screened using ICD10 and ICD9 diagnosis codes, including dementia combined with delirium, postoperative delirium, and other unspecified delirium cases, while alcohol and other psychoactive drug-induced delirium subtypes were omitted.

### Instrumental variables selection

We conducted several quality control steps to select eligible IVs. Candidate IVs from the GWAS results were included using a threshold of *p* < 1e^−5^ (27). The specific steps for selection were as follows: (1) To avoid linkage disequilibrium (LD), independent SNPs were retained adhering to criteria of *R*^2^ < 0.001, and window size of 10,000 kb. (2) The F-statistic, typically used to evaluate the strength of the correlation between each IV and exposure, where weak IVs with an *F* < 10 were excluded (28). The F-statistic was calculated using the formula 1: F = R^2^ (n-k-1)/k(1-R^2^), where *R*^2^ is the variance of exposure explained by the IV, n is the sample size, and k is the number of IVs. For metabolites, formula 2: F = (PVE(n-k-1))/(1-PVE) k was used, where PVE is the proportion of exposure variance for the IV. By setting k equal to 1, the PVE for each IV was calculated using the formula 3: PVE = (2*β^2^*MAF*(1-MAF))/[2*β^2^*MAF*(1-MAF) + 2*Se^2^*n*MAF*(1-MAF)] (25), where MAF is the minor allele frequencies. (3) Harmonizing the alleles and effects between the exposures and outcome and overlapped SNPs were excluded. (4) We searched SNPs with positive results using PhenoScanner and found no SNPs with potential confounding effects on delirium. (5) The Mendelian Randomization Pleiotropy RESidual Sum and Outlier (MR-PRESSO) (29) test was applied to identify potential horizontal pleiotropy. We mitigated the influence of pleiotropy by removing outliers, specifically SNPs with a global test *p* value under 0.05 in the MR-PRESSO test. Following removing these outliers, we conducted a re-analysis to ensure the accuracy of our results.

### MR analysis

We mainly focus on two relationships: between dietary component intake and delirium, and between blood metabolites and the risks of delirium. First, after rigorously screening quality IVs, a two-sample MR with at least three IVs was performed to explore potential causal relationships. The inverse-variance weighted (IVW) method was used as the primary MR analysis to estimate the causal effects. Then, other four methods were used for supplementary analyses, including the weighted median estimator (WME), maximum likelihood estimator (MLE), weighted mode-based estimator, and MR-Egger regression. Estimates are presented as odds ratio (OR) and 95% confidence intervals (CIs) for a single unit increment for each trait. False discovery rate (*FDR*) was used to adjust for multiple-testing correction. Results with *p* < 0.05 but above the *FDR* corrected significance threshold were considered suggestive evidence for a potential association.

To ensure the robustness of the results and to identify potential bias factors such as pleiotropy and data heterogeneity, we conducted additional sensitivity analysis. The sensitivity analysis encompassed a pleiotropy test, a heterogeneity test, and a leave-one-out method. MR-PRESSO method was used in the pleiotropy test, it was considered that the horizontal pleiotropy of the IVs would not significantly influence the causal inference if the intercept did not exceed 0.1 and the corresponding *p* value was above 0.05. The heterogeneity test was used to identify differences across the IVs. Additionally, the leave-one-out analysis was conducted to determine whether the MR estimate was driven or biased by a single SNP.

Finally, we performed reverse MR analysis on significant results to ensure the validity of the results and to avoid confusion in the causal interpretation.

### Software

Data cleaning and structuring were conducted using Jupyter Notebook in Python (version 3.0). All analyses were performed using the “TwoSampleMR” package and R Software (version 4.2.1).

## Results

### Selection of instrumental variables

The characteristics of the selected IVs for each dietary component intake are listed in Supplementary Table S1. The ultimate counts of 2,244 SNPs were selected as IVs for subsequent MR analyses between dietary component intake and delirium risks. Supplementary Table S2 provides the details of the selected IVs for each metabolite, and a total of 6,277 SNPs were selected as IVs in subsequent MR analyses.

In all analyses for delirium, the F-statistics of the IVs were > 10, indicating less possibility of weak instrument bias.

### Dietary component intake on delirium

**Figure 2:**
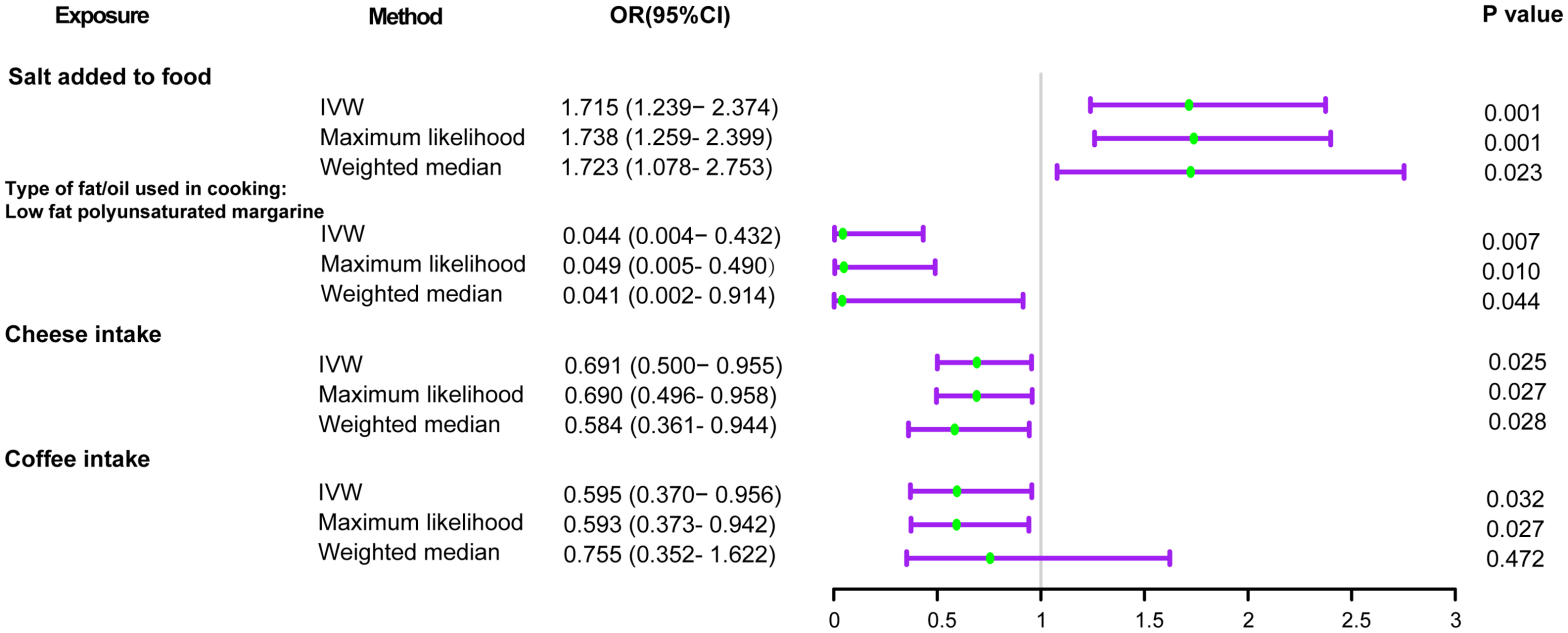
Forest plots of the MR results of dietary component intake with risks of delirium. OR, odds ratio; 95% CI, 95% confidence interval.

**Figure 3:**
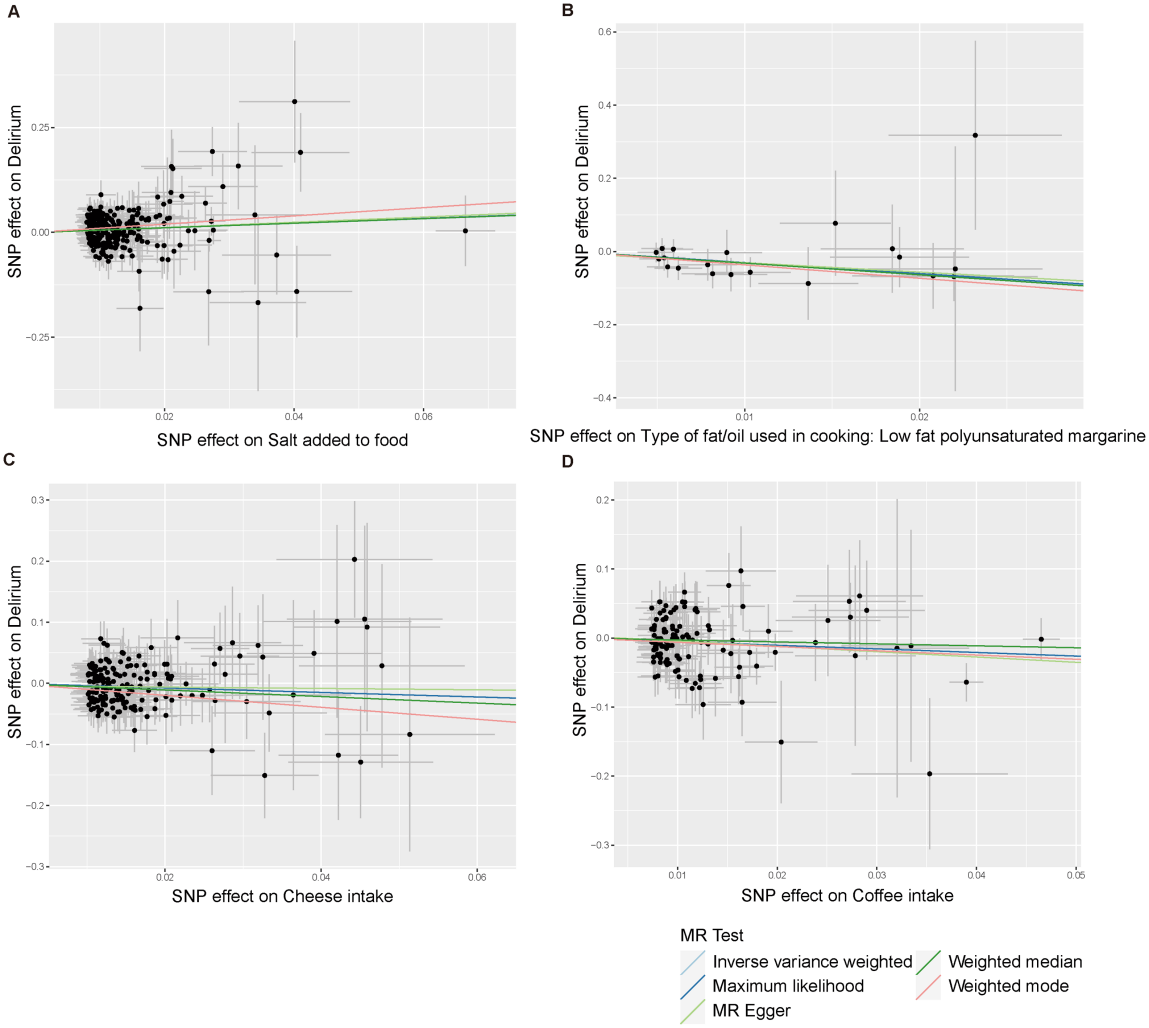
Scatter plots of the five MR models for causal relationships between four dietary component intake with risks of delirium. **(A)** Salt added to food. **(B)** Type of fat/oil used in cooking: low fat polyunsaturated margarine. **(C)** Cheese intake. **(D)** Coffee intake.

Using the IVW method (Figure 2), we found that host-genetic-driven salt added to food (OR _IVW_ = 1.715 [95%CI: 1.239–2.374], *p* = 0.001, *FDR* = 0.023) was significantly associated with a higher risk of delirium. There was evidence for a suggestive association between genetically predicted low-fat polyunsaturated margarine used in cooking (OR _IVW_ = 0.044 [95%CI: 0.004–0.432], *p* = 0.007) and cheese intake (OR _IVW_ = 0.691 [95%CI: 0.500–0.955], *p* = 0.025) with decreasing risk of delirium. We also found that genetically predicted coffee intake (OR _IVW_ = 0.595 [95%CI: 0.370–0.956], *p* = 0.032) was potentially associated with a lower risk of delirium. Consistent causal effects were observed for the above associations across different supplementary analyses including MR-Egger, MR-PRESSO, maximum likelihood, and weighted median methods, with similar directions and comparable magnitudes of effect (Figure 3 and Supplementary Table S3). We next performed heterogeneity and horizontal pleiotropy tests as sensitivity analyses and found no significant pleiotropy and heterogeneities (*p* > 0.05, Supplementary Table S3). Besides, the intercept of the MR-Egger regression approached 0, and the *p* value of the MR-Egger and MR-PRESSO global test was above 0.05, indicating no evidence of horizontal pleiotropy. The leave-one-out analysis showed the above-identified causal associations with no sensitivity to any single IVs (Supplementary Figures S1–S4).

### Blood metabolites on delirium

**Figure 4:**
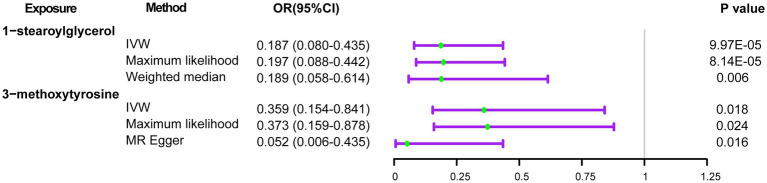
Forest plots of the MR results of blood metabolites with risks of delirium. OR, odds ratio; 95% CI, 95% confidence interval.

**Figure 5:**
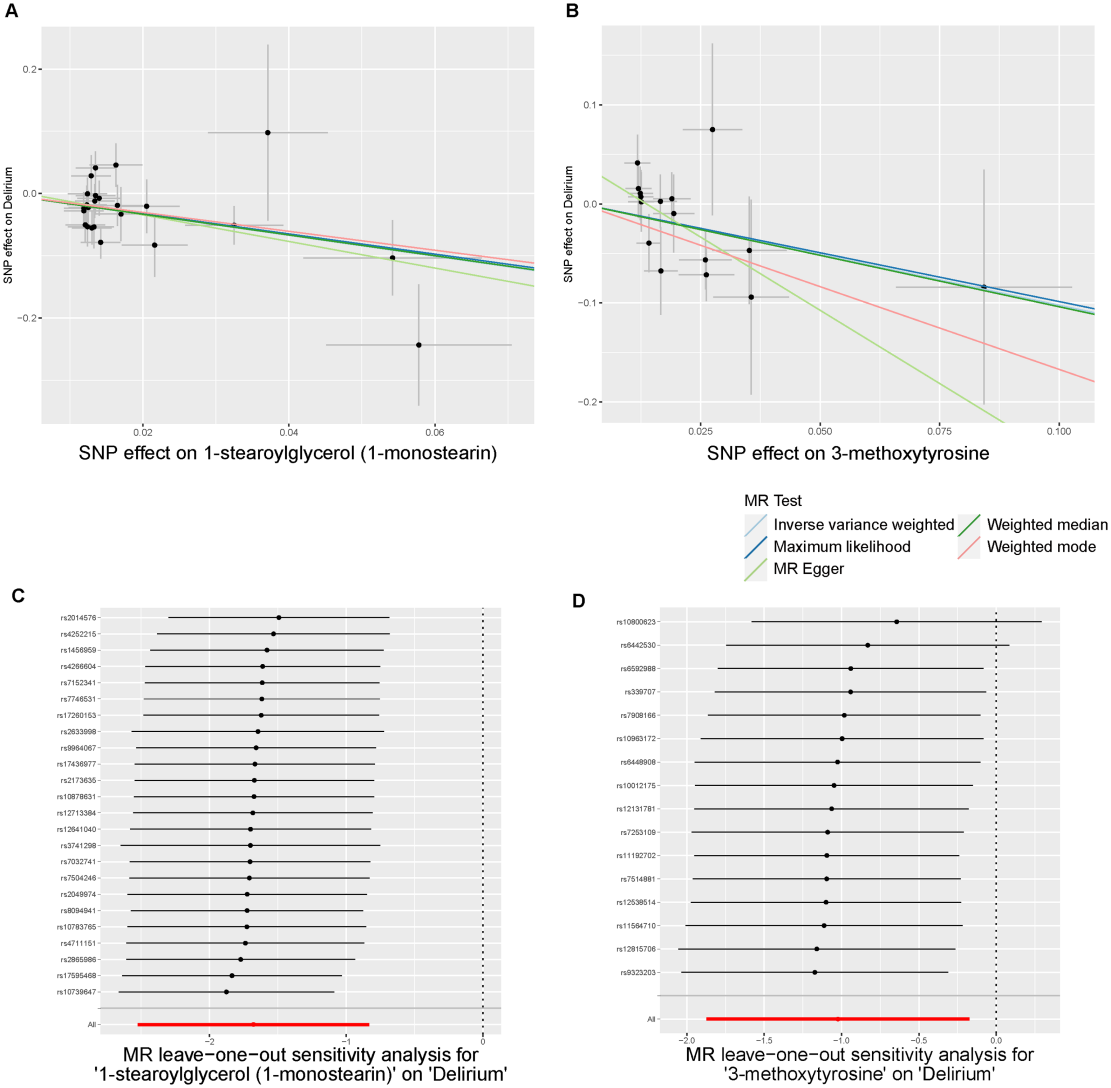
Scatter plots and Leave-One-Out plots of the MR results of blood metabolites with risks of delirium. **(A,B)** Scatter plots of the five MR models for 1-stearoylglycerol **(A)** and 3-methoxytyrosine **(B)** on delirium. **(C,D)** Forest plots of Leave-One-Out analysis results for 1-stearoylglycerol **(C)** and 3-methoxytyrosine **(D)** on delirium.

Initially, we identified eight annotated metabolites potentially to be associated with the risks of delirium (*p* < 0.05). Then, these associations are further validated using supplementary analyses. Finally, two metabolites with causal associations were validated in more than two MR methods (Supplementary Table S4). Genetically predicted 1-stearoylglycerol (OR _IVW_ = 0.187 [95%CI: 0.080–0.435], *p* = 9.97E-05, *FDR* = 0.025) was associated with decreased risks of delirium. Besides, the host-genetic-driven 3-methoxytyrosine (OR _IVW_ = 0.359 [95%CI: 0.154–0.841], *p* = 0.018) was also suggestive to be related to a decreased risk of delirium (Figure 4). Furthermore, sensitivity analyses showed no significant horizontal pleiotropy and heterogeneities (Supplementary Table S4). The scatter plots of the SNP effect sizes for the above associations are shown in Figures 5A,B, demonstrating relatively consistent effect direction and magnitude across methods. The leave-one-out sensitivity analysis confirmed that no individual SNPs significantly affected these associations (Figures 5C,D). Moreover, we further investigated whether there is a causal association between four types of dietary component intake related to delirium and 1-stearoylglycerol and 3-methoxytyrosine, and found no significant relationship (Supplementary Table S5).

In addition, we also investigated the causal relationship between some key metabolites related to low-fat polyunsaturated margarine used in cooking and coffee intake (30, 31) with the delirium risks (Supplementary Table S6). Given that low-fat polyunsaturated margarine is characterized by its low-fat content, we focused on blood lipid levels that might be affected by a low-fat diet. We found that lower levels of plasma levels of low-density lipoprotein cholesterol (LDL-C) are suggestively linked to a decreased risk of delirium (OR _IVW_ = 1.163 [95%CI: 1.008–1.342], *p* = 0.039). However, the four coffee-related metabolites did not show a causal relationship with delirium.

### Reverse MR

The results of the reverse MR analysis are shown in Supplementary Table S7. Here, no significant causal estimates were detected by the five MR methods, suggesting a lack of evidence for a causal effect from the delirium risks to identified dietary component intake or blood metabolites.

## Discussion

In our study, we performed an MR analysis to assess the causal impact of dietary component intake and blood metabolites on the risk of delirium. Our research provided evidence that the host-genetic-driven habit of salt added to food may significantly elevate the risk of delirium, while the habit of low-fat polyunsaturated margarine used in cooking, cheese intake, and coffee intake appears to mitigate the delirium risk. Furthermore, we found that the elevated level of genetically predicted 1-stearoylglycerol and 3-methoxytyrosine may contributed to reducing delirium susceptibility, suggesting a protective role against delirium development. The summary of the study is in Graphical abstract.

The recognition and management of delirium possess significant challenges for clinics, particularly within intensive care units (ICU) where it often goes under-recognized (8). While traditional management strategies have largely revolved around pharmacological treatments and multicomponent intervention (32), the potential of dietary interventions in reducing delirium risk is gaining traction thanks to their affordability, cost-effectiveness, and feasibility. Growing evidence suggests a causal link between dietary components and the risk of delirium, supplements like green tea polyphenols (33), fish oil omega-3 fatty acids (34), taurine (35), and melatonin (36) have been recognized for their beneficial roles in brain function, potentially aiding in the prevention of delirium (37). However, our understanding of how the habits of dietary component intake influence delirium development remains limited.

In this study, we provided evidence that specific dietary component intake is causally linked to delirium risk. Notably, we found that salt added to food with a notable 1.72-fold increase in delirium risk. Existing research has already established high salt intake as a primary risk factor for cardiovascular diseases, particularly through its association with hypertension. Furthermore, emerging evidence highlights its direct impacts on central nervous system (CNS) disorders and brain toxicity (38). For instance, diets high in salt have been shown to exacerbate cognitive decline and the development of Alzheimer’s disease-like pathology by influencing tau protein abnormalities, neurovascular unit dysfunction, and synaptic deficits (39, 40). Additionally, a high salt diet could induce stroke onset and brain toxicity involving sympathetic nerve activation and brain oxidative stress (41). It also impairs long-term brain recovery after stroke by modulating macrophage function and mitochondrial oxidative phosphorylation (42). Recent findings also suggested that high salt intake induced cognitive dysfunction and CNS autoimmune pathology by altering the gut-brain axis and promoting the differentiation of T helper 17 cells (43). Thus, we advocate that reducing the habit of adding salt to food may help decrease the risk of delirium.

Our study indicates that low-fat polyunsaturated margarine used in cooking, cheese, and coffee intake, might help lower delirium risks. Low-fat polyunsaturated margarine is rich in omega-3 polyunsaturated fatty acids (PUFA). The role of omega-3 PUFA supplementation is highlighted in delirium prevention, causing their neuroprotective, anti-inflammatory, and antioxidant properties (34, 44). However, our MR analysis does not confirm a direct causal link between omega-3 PUFA levels in blood and delirium. It suggests that omega-3 PUFA supplementation’s benefits may involve complex interactions beyond elevated blood omega-3 PUFA levels, possibly involving other nutrients or metabolic processes not fully captured in our analysis. Crucially, our study emphasized that low-fat, rather than normal-fat polyunsaturated margarine, reduces the risk of delirium. Moreover, our MR analysis also reveals a positive association between high levels of LDL-C and delirium risk, corroborated by an observational cross-sectional study that identifies LDL-C as a risk factor for postoperative delirium (45). Growing evidence suggests a link between elevated LDL-C levels and cognitive decline (46), possibly due to its role in cerebral vascular pathology. Thus, given the important role of low-fat diets and lowering LDL-C levels against cognitive decline and age-related disorders, opting for low-fat polyunsaturated margarine is advisable (47).

The health benefits of cheese intake were underscored, including its inverse associations with all-cause and cardiovascular mortality, dementia, and stroke incidence (48, 49). The healthy effects of cheese can be attributed to its rich content of high-quality proteins, minerals (e.g., calcium, phosphorus, and magnesium), vitamins (e.g., vitamin A, K2, B2, B12, and folate), probiotics, and bioactive molecules (e.g., bioactive peptides, lactoferrin, and milk fat globule membrane) (48). Cheese is a significant source of vitamin K2, known for maintaining neurocognitive functions by activating vitamin K-dependent proteins and aiding in sphingolipids synthesis (50). The matrix of cheese can help alleviate the adverse effects of saturated fat (51) and sodium-induced cutaneous microvascular dysfunction (50). Moreover, the lactic acid bacteria produced during cheese fermentation exert probiotic benefits in improving cognitive function and ameliorating neuroinflammation by modulating the gut-brain axis (52, 53). Thus, we speculate that the positive effects of cheese intake are likely due to the combined roles of various components rather than a single one. Further research is needed to identify the roles of these specific components or combinations.

Coffee has been recognized for its various pharmacological benefits, including anti-inflammatory, antioxidant, neuroprotective, and anticancer properties (54). Meta-analyses have consistently suggested that drinking three cups of coffee daily could prevent approximately 6% of years of healthy life loss (55). Furthermore, coffee consumption may offer protective effects against cognitive decline and depression (56, 57). Key neuroprotective compounds in coffee, such as caffeine, polyphenols, chlorogenic acid, and trigonelline (54), contribute to reducing pro-inflammatory cytokine release by microglia and promoting microglia phenotype shifting. Prophylactic use of caffeine perioperatively can prevent postoperative headaches (58) and facilitate quicker recovery from sedation and anesthesia, especially in those prone to post-extubation complications. However, clinical evidence on consuming coffee in perioperative periods remains scarce (58). Moreover, further studies are needed to explore which neuroprotective compounds in coffee, or their combination, might affect the risk of delirium.

Prior observational studies have established that metabolic imbalances play a significant role in the development of postoperative delirium, highlighting the involvement of ω3 and ω6 fatty acids, intermediates of the tricarboxylic acid cycle, and phosphatidylinositol (17, 18). Our MR results indicated that elevated blood 1-stearoylglycerol (1-SG) levels and 3-methoxytyrosine (3-MT) may causally contribute to reducing delirium risk. 1-SG, a lipid metabolite mainly converted to free fatty acids by monoacylglycerol lipase (MAGL), will accumulate due to reduced MAGL activity. Although still lacking direct evidence connecting 1-SG with delirium, both pharmacological or genetic inactivation of MAGL, have been demonstrated to alleviate neuroinflammation and neuropathology, and enhance synaptic and cognitive functions in animal models of neurodegenerative diseases (59, 60), suggesting a potential protective role against delirium. 3-MT is a major metabolite of dopa precursor L-dopa used in Parkinson’s disease treatment and has been shown to reduce brain dopamine (dopa) levels by limiting L-dopa breakdown (61). An increasing body of evidence points to neurotransmitter system dysfunctions, particularly the overproduction of acetylcholine, 5-hydroxytryptamine (5-HT), and dopa, as central to the pathophysiology of delirium (62, 63). Since the severe side effects of the first-line drug dopa receptor D2 antagonist haloperidol, psychiatrists are considering shifting toward alternative treatments targeting dopa and 5-HT (63). 3-MT attenuated the formation of dopa and 5-hydroxytryptophan (5-HT precursor) by about 25% in brain tissue (64). 5-HT receptor antagonist has demonstrated the potential to reverse delirium symptoms in rats (62). Thus, we speculated that 3-MT might correct dopa and 5-HT imbalances, which might help to prevent and treatment of delirium. However, further research is needed to clarify these metabolites’ roles in delirium and to identify effective metabolic-based interventions.

Our study presented several limitations. Firstly, the GWAS summary data primarily involved participants of European descent, potentially limiting the extension of our findings to other populations. Hence, future studies utilizing multiethnic cohorts are warranted to validate our findings. Additionally, this study provides new evidence for the causal associations between dietary component intake, blood metabolites, and delirium. Future experimental investigations are also needed to confirm the underlying biological mechanisms and to elucidate specific components or combinations that may mediate the effect of dietary intake on delirium.

## Conclusion

Our findings support a causal association between certain dietary component intake, blood metabolites, and delirium, and provide new evidence for potential intervention strategies for the prevention and treatment of delirium. Further research is warranted through randomized controlled trials (RCT) and experimental studies to elucidate the impact of specific dietary habits and metabolites on the development of delirium, including the mechanisms involved.

## Data Availability

The original contributions presented in the study are included in the methodology section of this article and Supplementary material, further inquiries can be directed to the corresponding authors.

## Footnotes

^1^https://biobank.ndph.ox.ac.uk/ukb/refer.cgi?id=113241

^2^https://biobank.ndph.ox.ac.uk/ukb/refer.cgi?id=118240

^3^https://gwas.mrcieu.ac.uk/datasets
