# Supplementary material for: The causal effects of dietary component intake and blood metabolites on risk of delirium: a Mendelian randomization study
Source: Front Nutr. 2024 Nov 27;11:1441821. doi: 10.3389/fnut.2024.1441821 (PMC11631601; doi:10.3389/fnut.2024.1441821)

Supplementary Figure 1. Forest plots of "Leave-One-Out" analysis for causal associations between salt added to food and risk of delirium.

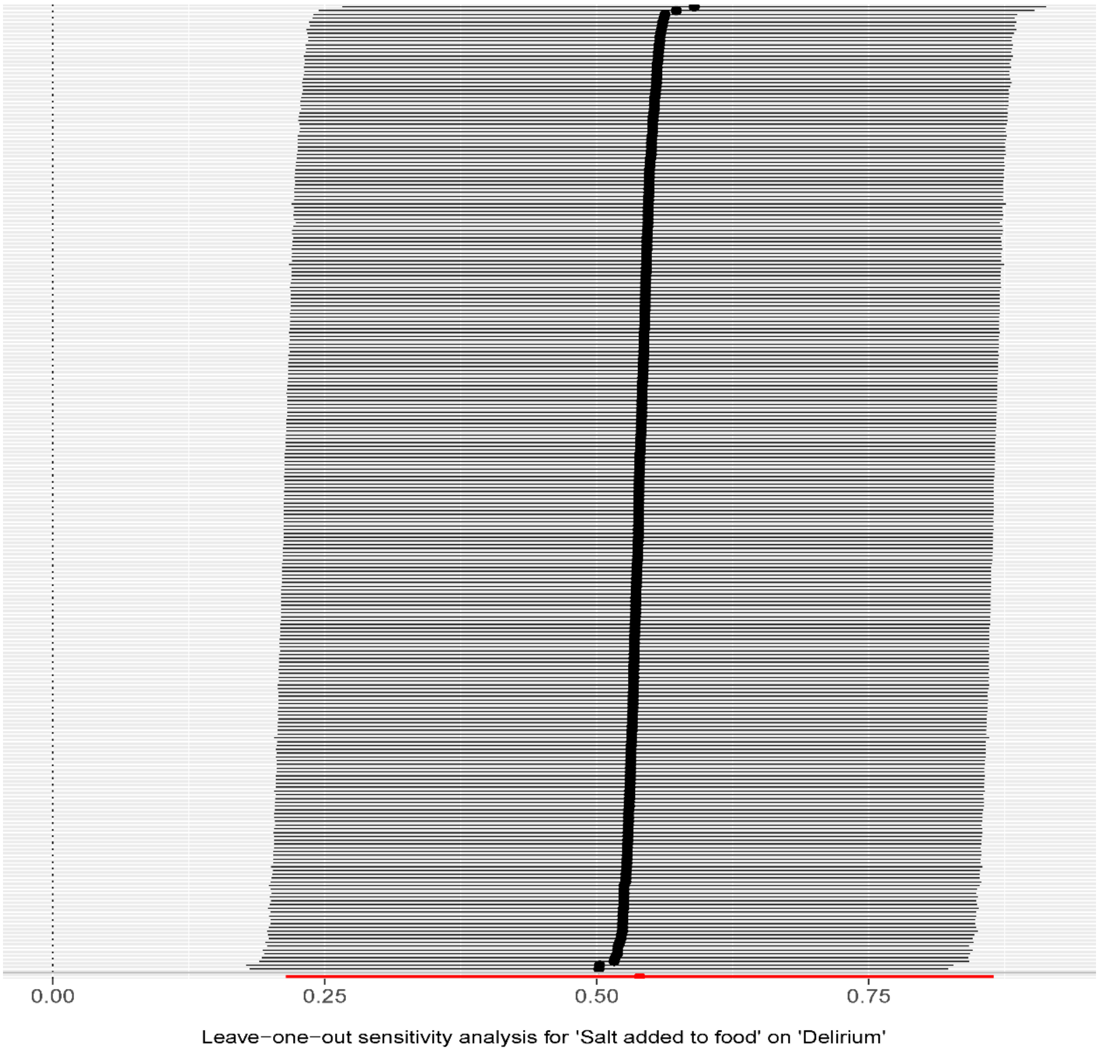

Supplementary Figure 2. Forest plots of "Leave-One-Out" analysis for causal associations between type of fat/oil used in cooking: low fat polyunsaturated margarine and risk of delirium.

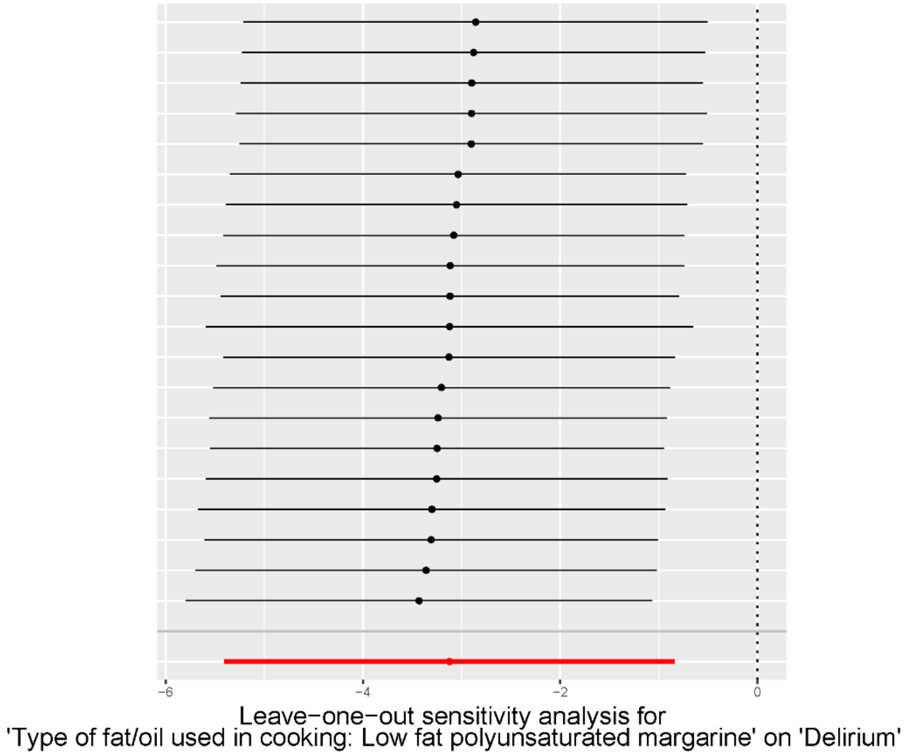

Supplementary Figure 3. Forest plots of "Leave-One-Out" analysis for causal associations between cheese intake and risk of delirium.

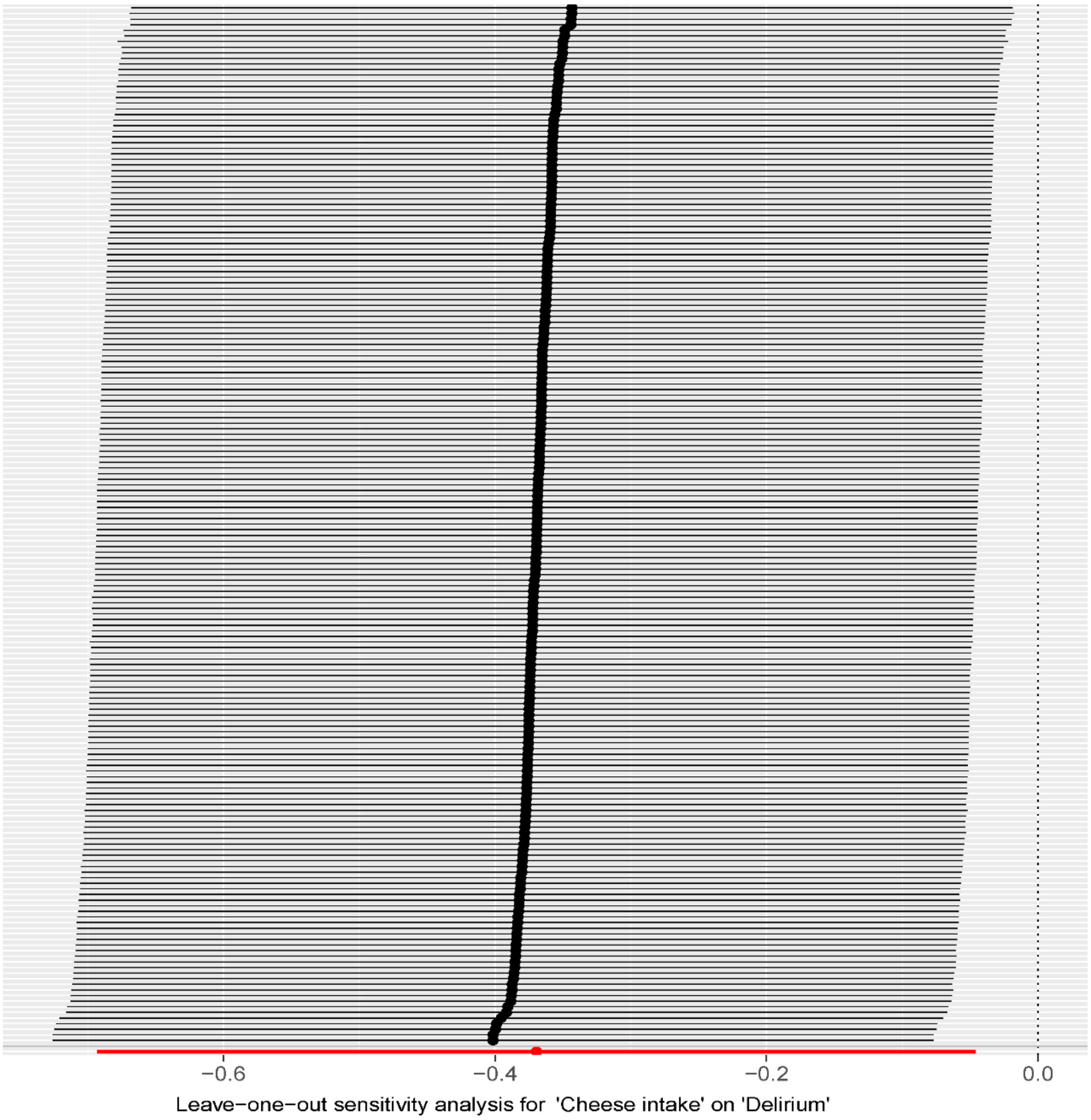

Supplementary Figure 4. Forest plots of "Leave-One-Out" analysis for causal associations between coffee intake and risk of delirium.

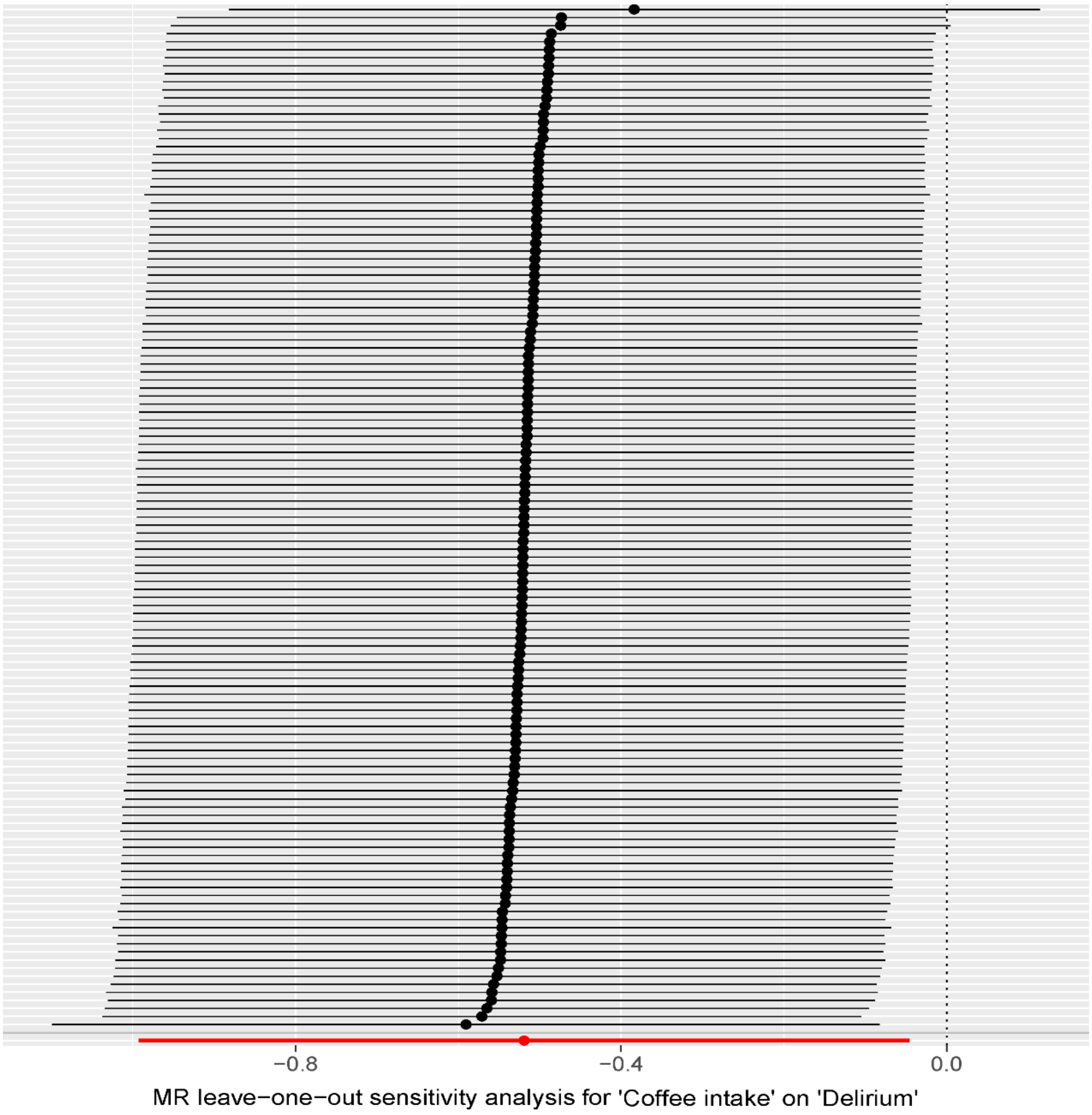

Supplement: Supplementary file 2 [file Image_1.pdf]
